# Progress in low-resolution *ab initio* phasing with *CrowdPhase*

**DOI:** 10.1107/S2059798316003405

**Published:** 2016-03-01

**Authors:** Julien Jorda, Michael R. Sawaya, Todd O. Yeates

**Affiliations:** a UCLA–DOE, Institute for Genomics and Proteomics, 611 Charles Young Drive East, Los Angeles, CA 90095, USA; b UCLA, Molecular Biology Institute, 611 Charles Young Drive East, Los Angeles, CA 90095, USA; c Department of Chemistry and Biochemistry, University of California, 611 Charles Young Drive East, Los Angeles, CA 90095, USA

**Keywords:** crowdsourcing, phase problem, *CrowdPhase*, direct methods

## Abstract

*Ab initio* phasing by direct computational methods in low-resolution X-ray crystallography is a long-standing challenge. A common approach is to consider it as two subproblems: sampling of phase space and identification of the correct solution. While the former is amenable to a myriad of search algorithms, devising a reliable target function for the latter problem remains an open question. Here, recent developments in *CrowdPhase*, a collaborative online game powered by a genetic algorithm that evolves an initial population of individuals with random genetic make-up (*i.e.* random phases) each expressing a phenotype in the form of an electron-density map, are presented. Success relies on the ability of human players to visually evaluate the quality of these maps and, following a Darwinian survival-of-the-fittest concept, direct the search towards optimal solutions. While an initial study demonstrated the feasibility of the approach, some important crystallographic issues were overlooked for the sake of simplicity. To address these, the new *CrowdPhase* includes consideration of space-group symmetry, a method for handling missing amplitudes, the use of a map correlation coefficient as a quality metric and a solvent-flattening step. Performances of this installment are discussed for two low-resolution test cases based on *bona fide* diffraction data.

## Abstract 2

New developments in *CrowdPhase*, a collaborative online game for tackling the low-resolution phase problem, are presented. The new features address several crystallographic issues and extend the reach of *CrowdPhase* to a broader range of experimental data sets.

## 1. Introduction   

In just a few years, crowdsourcing and gamification have become important actors in efforts to solve challenging scientific problems, owing in part to the emergence of cloud computing and social networks. Crowdsourced initiatives such as *Foldit* (Khatib, Cooper *et al.*, 2011 ▸; Khatib, DiMiao *et al.*, 2011 ▸), *EteRNA* (Lee *et al.*, 2014 ▸) and numerous others (Gardner *et al.*, 2011 ▸; Kelder *et al.*, 2012 ▸; Loguercio *et al.*, 2013 ▸) are convincing examples illustrating that, in certain cases, nontrivial scientific problems can be subdivided into elementary tasks and effectively distributed to a collective workforce. Along these lines, we recently demonstrated that the pattern-recognition abilities of a group of players could be harnessed to attack the low-resolution phase problem in X-ray crystallography (Jorda *et al.*, 2014 ▸). Specifically, we involved non-expert users in a collaborative game called *CrowdPhase* in an attempt to determine *ab initio* the best sets of phases for low-resolution data sets. At its core, *CrowdPhase* is driven by a modified genetic algorithm that evolves a population of candidate solutions. Each candidate solution (or individual) comprises a set of phases for the observed reflections; in the language of genetic algorithms, each phase is a gene in the complete genome of an individual. Each individual presents a unique phenotype, here manifested in the form of an electron-density map.

The stochastic exploration of diffraction phase space has been widely discussed in various contexts (Baker *et al.*, 1993 ▸; Colovos *et al.*, 2000 ▸; Holton *et al.*, 2000 ▸; Miller *et al.*, 2001 ▸; Terwilliger, 2001 ▸; Webster & Hilgenfeld, 2001 ▸; Lunina *et al.*, 2003 ▸; Abdurahman & Purwanto, 2008 ▸; Dumas & van der Lee, 2008 ▸; Uervirojnangkoorn *et al.*, 2013 ▸). The exploration of phase space at low resolution (where the number of reflections to phase is typically small) has been undertaken in a number of previous studies aimed at achieving *ab initio* phasing. A number of map-evaluation criteria have been investigated for their utility in identifying good phase sets, including analysis of electron-density histograms (Lunin, 1993 ▸), map connectivity (Baker *et al.*, 1993 ▸; Lunin *et al.*, 2000 ▸), topological features (Fokine *et al.*, 2003 ▸), maximization of likelihood (Petrova *et al.*, 1999 ▸, 2000 ▸) or figure of merit with atoms approximated as Gaussian scatterers (Lunin *et al.*, 1995 ▸). Solutions with some of these techniques have been reported with varying degrees of success, including the low-resolution phasing of human paraoxonase (Fokine *et al.*, 2003 ▸), RNase *sa* (Lunin *et al.*, 2000 ▸), an AspRS–tRNA^Asp^ complex (Lunin *et al.*, 1995 ▸) and Na^+^-NQR (Lunin *et al.*, 2013 ▸). What differentiates *CrowdPhase* from these techniques is the gamification of the problem and the use of collective human intelligence as a fitness function to guide the optimization process. In practice, the human decisions are integrated into the genetic algorithm workflow at two levels. The main contribution occurs during the tournament step, where each player decides the outcome of a tournament by selecting the two ‘fittest’ individuals from a large set of maps based on their visual features; players are subsequently invited at the very end of the evolutionary process to pick the best individual from the last generation, which we refer to as the termination step.

In the initial work, we devised two synthetic phasing puzzles in which the phases were known from a model and perfect (calculated) structure-factor amplitudes were assumed. This allowed us to assess the capability of the human-powered genetic algorithm in highly idealized situations. The first puzzle involved two C atoms extracted from an arbitrary structure in the PDB (Miller *et al.*, 1996 ▸), while the second corresponded to a six-stranded β-barrel structure referred to as cylindrin (Laganowsky *et al.*, 2012 ▸). The data sets were expanded to a synthetic *P*1 unit cell and restricted to resolutions of 25 Å for the two-atom puzzle and 18 Å for the cylindrin, leading to final data sets with 37 and 67 reflections (or genes) to phase, respectively. In both cases, *CrowdPhase* was able to arrive at good phase sets based on the players’ choices, and low-resolution molecular boundaries were obtained in agreement with the true solutions. While these results constituted a proof of concept that crowdsourcing can be used to obtain *ab initio* phases at low resolution in ideal situations, several crystallographic concerns were not considered at the time of the initial study. Further developments were necessary to account for crystal symmetry (*i.e.* non-*P*1 space groups), the existence of unmeasured data (*e.g.* at low resolution) and nonzero bulk-solvent density in real-case scenarios. Here, we present improved treatments of these issues in *CrowdPhase* and discuss the results obtained when using this new version to solve two puzzles that were built on true experimental data.

## 2. Materials and methods   

### 2.1. Space-group symmetry implementation   

The set of symmetry operators for each space group was represented by transformation matrices, and a list of equivalent origin shifts for map (and phase) comparisons was incorporated. Information relating to space-group symmetry comes under consideration at two levels: the Fourier transform and our custom map-alignment method (Jorda *et al.*, 2014 ▸).

### 2.2. Unmeasured amplitudes   

Particularly in the lowest resolution range, diffraction data sets often include a number of unmeasured reflections. Given the relatively small number of total reflections under consideration in the present work, these missing reflections can dramatically affect the resulting electron-density map if not addressed. To circumvent this problem, we developed a method in which these amplitudes are considered as additional genes to be explored, similar to the phases. In our framework, the discrete values that a short binary string can encode are scaled by multiplication to give a set of possible structure-factor amplitudes. In this case, a three-bit binary string can return any integer between 0 and 7, where 0 corresponds to a decoded amplitude of 0 and 7 is the maximal amplitude allowed for the studied case. To determine this maximal amplitude, we calculate a Wilson plot and fit a least-squares regression line through ln(*F*_obs_)^2^ against sin^2^(θ)/λ^2^ to the data. The intercept of this regression line has a dual use in *CrowdPhase*: inferring the value of the maximal amplitude as four times the intercept and a scale factor to put the amplitudes on an absolute scale for later comparison with the structure factors of the true model. Upon defining this maximal amplitude, the gene is initialized with a random binary string and is subsequently submitted to the canonical genetic algorithm evolutionary procedure.

### 2.3. Solvent flattening   

Density-modification methods typically involve a cyclic protocol that alternates between modifications of the electron-density map in real space and a combination of the initial structure-factor amplitudes with the modified phases. In the original solvent-flattening procedure proposed by Wang (1985 ▸), the electron density at each grid point is replaced by the weighted average density of grid points that are within a sphere of radius *R*. The resulting map is subsequently Fourier-analyzed and the phases of the calculated structure factors replace or modify those before the real-space modification. Our adaptation of solvent flattening differs in two points. The modification is performed directly in reciprocal space following published equations (Leslie, 1988 ▸) and involves only one cycle. For unmeasured reflections, the calculated amplitudes and phases from the density-modification step are both retained. The radius of the sphere used to apply the weighting function is defined in *CrowdPhase* as twice the grid spacing of the map.

### 2.4. Map correlation coefficient   

The map correlation coefficient is a measure commonly used in model building or refinement for phase comparison. While a simple approach would be to operate at the level of real space and consider the correlation of the density for each grid point (Read, 1986 ▸), applying the procedure to each electron-density map newly generated by *CrowdPhase* is cumbersome because of its space and time complexity. For this reason, we preferred the implementation of the map correlation calculation as defined by Lunin & Woolfson (1993 ▸), which operates at the level of reciprocal space and involves only structure factors.

## 3. Results   

### 3.1. Crystallographic improvements   

One of the first modifications in *CrowdPhase* was to integrate the handling of crystallographic symmetry. In our initial study, structure factors were expanded to an artificial unit cell in *P*1, thus avoiding symmetry calculations in the original pipeline (Jorda *et al.*, 2014 ▸). To extend the approach to higher order symmetry data sets, we enabled in this new installment the possibility of implementing, for any space group, its corresponding crystal lattice, symmetry operators and alternate origins (and corresponding phase shifts) that need to be considered when comparing two phase sets. The second practical extension was to implement the use of observed structure-factor amplitudes for phasing instead of those calculated from a model. However, a challenge arose from the fact that experimental data sets typically present at least a few unmeasured reflections, which, if ignored, could significantly affect the resulting electron-density maps. To address this problem in the context of our genetic algorithm, we considered the unobserved amplitudes as additional genes whose values needed to be optimized during the search. We represent the unobserved amplitudes as genes by encoding them as binary strings, following the formalism established for the phases. While the inclusion of missing amplitudes expanded the size of the search space, we were able to partially offset this increase by reducing the number of bits in the binary string for each phase to three (or one for centric reflections); the nine bits per phase in our initial study were judged unnecessary. Another significant change was made in the monitoring of the fitness or phase quality of generated individuals. Our initial study relied on the weighted r.m.s. phase error, but this measure of agreement does not lend itself to assessing the correctness of estimated amplitudes for unobserved reflections. For this reason, we replaced the weighted r.m.s. phase error with the map correlation coefficient as defined by Lunin & Woolfson (1993 ▸), a commonly used metric for comparing phases and electron-density maps. One last modification was to improve the molecule-like features of each new individual. We implemented a one-pass solvent-flattening step (Wang, 1985 ▸; Leslie, 1988 ▸), a density-modification procedure that is routinely used for improving the quality of maps and delineating molecular boundaries. The flattening was applied to the electron-density map of each newly generated individual after the usual genetic operations used to combine the genes (or phase sets) of two parents. A Fourier transform of the flattened map yields modified phases that are assigned to the new individual. A final improvement was brought to the definition of the contouring level of the displayed maps. Instead of fixing this level at a defined contour value (*e.g.* 1σ in previous studies), we adjusted the contouring based on the solvent:protein content ratio in the unit cell, ensuring consistency of the displayed volume in all maps.

### 3.2. Other improvements   

During our monitoring of games in *CrowdPhase*, we noticed a harmful side effect of the gamification: players that do not perform well tend to play more rounds than others in an attempt to keep their scores up with the other players in competition. In several cases, this degraded the overall performance. Another important issue was that a highly active player could play an unlimited number of tournament rounds in a single generation, allowing single users to evolve whole generations on their own. To mitigate these potentially detrimental influences, we established a credit system that restricts each player to 20 credits (or tournament rounds) per generation; a new generation is filled up typically by a total of 120 tournaments, each producing one new individual.

To assess the newly implemented features, two games aiming at phasing *ab initio* cases with higher order symmetry were run in *CrowdPhase*: the first puzzle was based on the crystal structure of the *Cucumber necrosis virus* capsid (PDB entry 4llf; Li *et al.*, 2013 ▸), while a second involved a racemic mixture of the synthetic cyclic peptide kalata B1 (kB1; PDB entry 4ttm; Wang *et al.*, 2014 ▸). As was the case in previous puzzles, the true phases were taken as known quantities for the sake of evaluating the quality of the phasing as the generations progressed.

### 3.3. The viral capsid puzzle   

**Figure 1:**
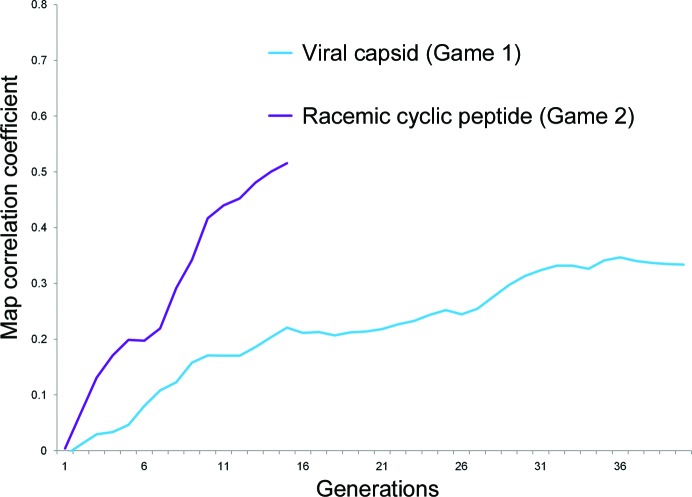
Monitoring the phasing evolution across generations for two low-resolution experiments in *CrowdPhase*. The plot shows the evolution of the average map correlation coefficient at each generation for the viral capsid puzzle (blue) and the centrosymmetric polypeptide puzzle (purple). The first game reached its termination step at the 40th generation, while the centrosymmetric experiment ended at generation 15.

**Figure 2:**
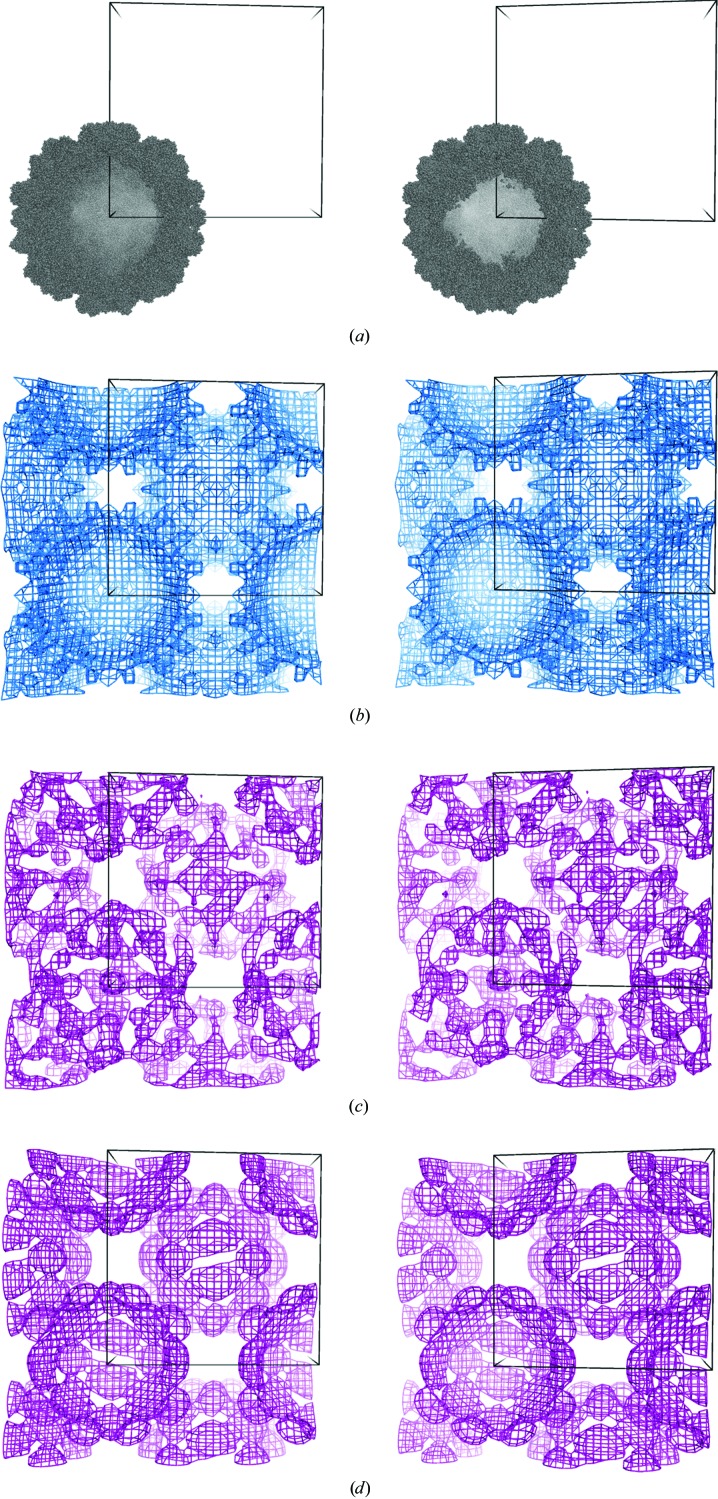
Stereoviews of electron-density maps derived from the viral capsid game. The atomic model of the viral capsid is represented sitting at one corner and the center of the centered cubic unit cell (*a*). The electron-density map calculated using model phases is depicted as a blue mesh in (*b*). The electron-density map obtained with *CrowdPhase* was calculated by combining the observed amplitudes with predicted phases and amplitudes and is shown in purple (*c*); (*d*) represents the same map after NCS averaging. All electron-density maps were displayed with a contour level of 1σ.

The viral capsid structure is in the cubic space group *I*23, with two capsids in the centered unit cell and a solvent content of 68% (Fig. 2*a*). We treated this problem at 45 Å resolution, where there are 70 possible reflections. All reflections had their unknown phases encoded in the genome of each individual, while 38 of the genes were reflections that were not observed during data collection and had their unknown amplitudes encoded in the genome as well. This operation extended the total number of genes to 108 in this case. The genetic algorithm parameters were defined as follows: 120, 12, 0.2, 0.3 and 40 for the population size, tournament size (the number of randomly chosen individuals presented to a player for the selection of two parents), mutation rate, crossing-over rate and the generation number for the termination step, respectively. For this game, 78 participants, mostly non-expert students, were invited to evolve the population of individuals with initially random phase sets after being given instructions on the shape they might expect, in this case a roughly spherical viral capsid. Of these 78, 52 passed the training step defined in our earlier work (Jorda *et al.*, 2014 ▸); this required some ability to select maps that were better than random. At the 40th generation, the average map correlation of the whole population reached a value of 0.33 when compared with the true solution (Fig. 1 ▸), while the fittest individual from this population featured a map correlation of 0.38. Additionally, the standard deviation between the set of map correlation values at each generation decreased steadily until reaching a value of around 0.03 in the final generation. As a final selection step, the players were asked to pick the individual that they judged to be the most fit from this final generation. The map chosen by the majority exhibited a correlation of 0.37 with the true solution (Figs. 2 ▸ *b* and 2 ▸ *c*). A very rough approximation of the capsid structure appeared at the correct location.

As a further step, we evaluated whether this phase set, obtained *ab initio* from crowdsourcing, could be improved by noncrystallographic symmetry (NCS) averaging. In applicable cases NCS can serve as a powerful constraint in phasing (Rossmann & Blow, 1962 ▸; Bricogne, 1976 ▸); the required routines have been implemented in widely used crystallo­graphic programs (Jones, 1992 ▸; Terwilliger, 2003 ▸; Cowtan *et al.*, 2012 ▸). Previous studies on viral capsid structures at low resolution have demonstrated that phase sets, either experimental or generated *ab initio*, could have their quality substantially improved by NCS averaging (Winkler *et al.*, 1977 ▸; Luo *et al.*, 1987 ▸; Rossmann, 1995 ▸; Miller *et al.*, 1996 ▸). In this case, we applied a round of iterative 15-fold NCS averaging (the internal symmetry arising from the icosahedral fivefold symmetry combined with a *T* = 3 triangulation) to the final map chosen by the *CrowdPhase* players using *AVE* (Jones, 1992 ▸). The resulting map showed a marked improvement visually, a map correlation of 0.75 with a map based on correct phases and very clear agreement with the known structure (Fig. 2 ▸ *d*). As a control, we also applied the same NCS averaging procedure to ten randomly phased starting map. The mean starting correlation for these maps was 0.001 and the mean final correlation was −0.20, with a standard deviation of 0.45; these maps generally appeared noisy.

Finally, in order to evaluate how this result compares with automated methods, we tried to phase the viral capsid data set using a spherical-shell model following the protocol described in Tsao *et al.* (1992 ▸). As a starting point, we positioned a uniform spherical density of 155 Å radius on the origin as an initial approximation of the envelope of the *Cucumber necrosis virus* capsid. The correlation of the map to the correct solution began at 0.34 for the spherical model and increased to 0.45 after 100 cycles of solvent flattening. An additional step of NCS averaging identical to that described above brought the map correlation to 0.68. This value is only slightly inferior to the value yielded by the *CrowdPhase* solution, but we note that the starting point for the *CrowdPhase* study was random phases, whereas the starting point in this latter comparison benefited from being able to unambiguously identify the origin of the capsid in advance and assign a spherical starting shape; these advantages would not be available in a more typical problem.

### 3.4. The cyclic peptide puzzle   

**Figure 3:**
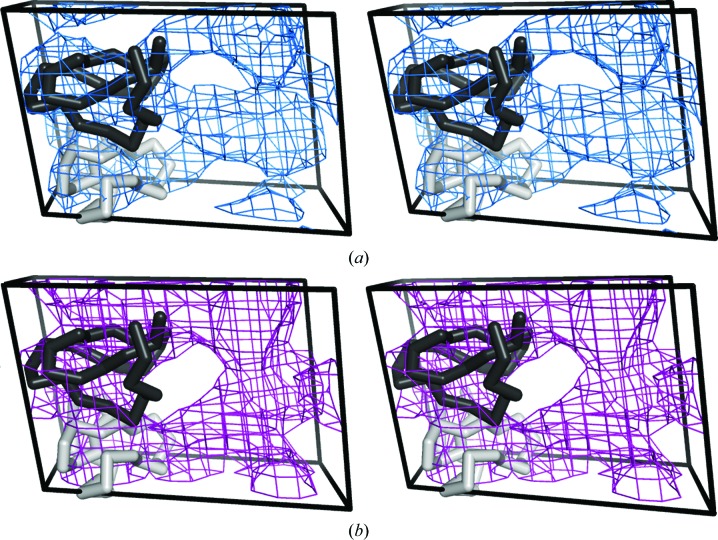
Stereoviews of electron-density maps for the centrosymmetric data set. The electron-density map calculated with model phases is depicted in blue (*a*), the map calculated using phases obtained from *CrowdPhase* is in purple (*b*) and an atomic model of the molecules found in one asymmetric unit is shown in gray. We defined the contour level at 0.6σ in both cases.

As a second test, we sought to assess the ability of *CrowdPhase* to phase a structure in a centrosymmetric space group; the prospects for determining structures of (synthetic) racemic mixtures of d and l polypeptides and small proteins have been discussed previously (Zawadzke & Berg, 1992 ▸, 1993 ▸; Hung *et al.*, 1998 ▸; Yeates & Kent, 2012 ▸). In this case, we used diffraction data collected from crystals of a racemic mixture of the 29-residue cyclic polypeptide abbreviated kB1 (Wang *et al.*, 2014 ▸). The centrosymmetric crystal belongs to space group 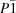 and has a solvent content of 31%. From this data set, 43 structure factors were calculated to 10 Å resolution and the genetic algorithm formalism was used to encode their phases. In this case, the parameters of population size, tournament size, mutation rate, crossing-over rate and the terminal generation number were set to 80, 12, 0.2, 0.3 and 15, respectively. The gene size was also restricted to a single bit, a representation that is sufficient to account for the two phase values (0 and 180°) allowed by the centrosymmetric space group. This game was played by 43 noncrystallographers with only a rough description of the molecule of interest (*i.e.* its size and its cyclic nature). Among the 43 initial players, 21 reached the required level to participate actively in the game. This game showed clear improvement over the random starting point. The evolution of the map correlation is shown in Fig. 1 ▸, where the average map correlation of the population consistently improves through the 15th generation, the value defined by the preset termination condition. Here again the standard deviation of the set of map correlation values across individuals in a generation steadily decreased from 0.2 to a value of around 0.04, at which point the population had begun to converge. Individuals of the final generation embodied phase information that was significantly better than the initially random phases. Indeed, the average map correlation of the whole population was 0.51, while the best individual of this generation featured a correlation of 0.61 (Fig. 1 ▸). As in the viral capsid game, players were asked to vote for a winning map. The chosen solution featured a correlation of 0.52 compared with the correct map based on model phases (Fig. 3 ▸). In the final solution the rough boundary is evident, although more detailed features are not.

Similarly to the previous puzzle, we sought to assess the performance of *CrowdPhase* by exploring the phasing of this centrosymmetric problem using existing methods. We first tried to obtain phases using *SHELXD* (Sheldrick, 2010 ▸). This program generally requires high-resolution data for success, and as a control we tested the ability to phase the given structure at 1 Å resolution (using synthetic calculated structure-factor magnitudes). This phasing test at 1 Å was successful in producing an essentially correct structure. We then attempted to obtain phases at the low resolution of our test problem (30 reflections extending to 10 Å). As anticipated, the program was not able to identify a correct solution at such low resolution. This prompted us to evaluate a previously developed program (*GENMEM*) specifically designed for *ab initio* low-resolution phasing based on map connectivity (Lunina *et al.*, 2003 ▸). The *GENMEM* program was used to generate 5000 phase sets, of which the top 100 were averaged by the program *AVERAGE*, which is also provided in the *GENMEM* package. In this case, the map correlation obtained between the resulting data set and the true solution was only 0.14.

## 4. Discussion   

A series of additions and improvements have been made to the *CrowdPhase* program. One of the main modifications was the consideration of crystal symmetry, enabling the system to handle a complete range of problem cases. Additional modifications such as the special treatment of unmeasured reflections, a solvent-flattening step and a map correlation coefficient were also implemented, with similar objectives.

To evaluate the new *CrowdPhase* functionalities, we designed two games involving higher order symmetry and analyzed their results following a similar protocol as in the former version (Jorda *et al.*, 2014 ▸). The first puzzle was based on a roughly spherical viral capsid, while the second one was a racemic mixture of a cyclic polypeptide and its enantiomer. The two games were initialized with essentially identical genetic algorithm parameters, with the exception of the termination step, which was set to generation 40 and 15 for the viral capsid and the centrosymmetric case, respectively. Overall, both game scenarios led from a random starting point to a visible improvement in the phases and the map correlation coefficients, especially in the early stages of the evolutionary process (Fig. 1 ▸).

In the viral capsid game, there was already substantial agreement between the correct map and that chosen by *CrowdPhase* players (Fig. 2 ▸). However, given the general ability of NCS averaging to improve the quality of phasing for problems with higher internal symmetry such as icosahedral viruses, we sought to determine whether the phase quality of the winning map in the viral capsid game could be improved. Substantial improvement was evident after NCS averaging, resulting in a map in which the essential features of the viral capsid were clear (Fig. 2 ▸ *d*). For comparison, an NCS averaging procedure starting from random phase sets (but without phase evolution by *CrowdPhase*) did not on the whole lead to significant improvement. Some starting phase sets did show improvement, while others were made worse by averaging (an outcome possibly related to the formation of Babinet negated images, as discussed previously; Miller *et al.*, 2001 ▸). Additional comparison of our results with a spherical shell *ab initio* approach relying on prior knowledge of the capsid origin showed that *CrowdPhase* produced a map with slightly better agreement with the true solution.

Likewise, the best map of the last generation in the racemic polypeptide game recapitulates some of the map features found in the correct solution (Fig. 3 ▸). *CrowdPhase* succeeded in delivering phase sets that were well correlated with the true solution in only 15 generations, which represented a more rapid convergence compared with the case of the viral capsid. This is a result that we had anticipated as this case was of lower complexity; a lower number of reflections, and fewer bits to describe the centric phases, made this second problem more tractable. The phase improvement in this case also exhibited an approach to a limiting value, although this point may not have been fully reached in the game conducted; some incremental gains may have been possible before the final plateau (Fig. 1 ▸). Here again, a comparison with existing *ab initio* phasing methods demonstrated that *CrowdPhase* was able to achieve greater improvements starting with random phases.

The plateau behavior observed in these studies, especially the case of the viral capsid, suggests that human visualization is better at discriminating totally spurious maps and is less capable of selecting better maps among a progressively improving set of maps. This is a trend that we have already discussed in the context of the minimal phase difference to preserve the discriminatory power of the human eye (Jorda *et al.*, 2014 ▸). In these two cases, using the map correlation as a metric for map comparison, the stagnation effect seems to happen when the standard deviation of the map correlation coefficient values falls below about 0.04. Hence, while the fitter choices are evidently identifiable among randomly generated individuals during the initial steps, the task becomes more challenging as the genetic algorithm progresses. The large proportion of users who succeeded in levelling up from the training phase in the first game (52 out of 78 players) supports this idea.

User training is another consideration that may relate to the degradation of the accuracy in the fitness evaluation along the evolutionary process. In these new studies, two drawbacks in the current training scheme were evident. Firstly, new users entering a game are trained on data (*i.e.* electron-density maps) that are related to the generation being examined by *CrowdPhase* at that point in time. For example, a user entering the game at generation 10 will be trained on maps from that generation. However, after such a user develops successful visual rules for identifying good maps, those rules might not hold up in later generations. The second issue comes from the fact there is no guidance for users besides the scoring function, which currently relies on the existence of a known solution. We plan to address the first problem by offering several short training sessions, not necessarily based on the current data, during the whole process rather than just one at the beginning. A second improvement would consist of the design of a more generalized training guide independent of any specific game data in *CrowdPhase*. A first step towards this goal was offered during the racemic peptide game, where users were presented with maps with different amounts of phase error based on the crystal structure of a different cyclic peptide.

In terms of new directions, we are currently working on new metrics that could guide human decisions based on computational analysis of topological and geometrical features of electron-density maps, following ideas introduced in an earlier study (Colovos *et al.*, 2000 ▸). Within our *CrowdPhase* framework, such a metric could be implemented as the sole fitness function for automatic phasing or as a guide combined with human visual discrimination. Future developments also include methods that would extend the *ab initio* phasing approach to higher resolution. These upcoming modifications combined with the presented crystallographic improvements should advance *CrowdPhase* as a potentially useful approach for low-resolution *ab initio* phasing.
